# Lateral Flow Aptasensor for Simultaneous Detection of Platelet-Derived Growth Factor-BB (PDGF-BB) and Thrombin

**DOI:** 10.3390/molecules24040756

**Published:** 2019-02-20

**Authors:** Guodong Liu, Anant S Gurung, Wanwei Qiu

**Affiliations:** 1 Institute of Biomedical and Health Science, School of Life and Health Science, Anhui Science and Technology University, Chuzhou 233100, China; qiuwanweiqww@126.com; 2 Department of Chemistry & Biochemistry, North Dakota State University, Fargo, ND 58105, USA; anant.gurung@ndsu.edu

**Keywords:** lateral flow, aptamer, aptasensor, PDGF-BB, thrombin

## Abstract

Here we report a lateral flow aptasensor (LFA) for the simultaneous detection of platelet-derived growth factor-BB (PDGF-BB) and thrombin. Two pairs of aptamers, which are specific against PDGF-BB and thrombin, respectively, were used to prepare the LFA. Thiolated aptamers were immobilized on a gold nanoparticle (AuNP) surface and biotinylated aptamers were immobilized on the test zones of an LFA nitrocellulose membrane. The assay involved the capture of PDGF-BB and thrombin simultaneously in sandwich-type formats between the capture aptamers on the test zones of LFA and AuNP-labeled detection aptamers. AuNPs were thus captured on the test zones of the LFA and gave red bands to enable the visual detection of target proteins. Quantitative results were obtained by reading the test band intensities with a portable strip reader. By combining the highly specific molecular recognition properties of aptamers with the unique properties of lateral flow assay (low-cost, short assay time and a user-friendly format), the optimized aptasensor was capable of simultaneously detecting 1.0 nM of PDGF-BB and 1.5 nM of thrombin in association with a 10-min assay time. The biosensor was also successfully applied to detect PDGF-BB and thrombin in spiked human serum samples. The LFA shows great promise for the development of aptamer-based lateral flow strip biosensors for point-of-care or for the in-field detection of disease-related protein biomarkers.

## 1. Introduction

The detection and the quantification of proteins play pivotal roles in basic discovery research and clinical applications [1]. Thrombin is an extracellular serine protease that plays a crucial role in the blood coagulation cascade, thrombosis, and hemostasis [2,3]. Its concentration in blood varies from nanomolar to low micromolar levels. The concentration of thrombin is connected to various coagulation abnormalities [4]. Platelet-derived growth factor (PDGF) is an important growth factor protein in human platelets that regulates cell growth and division toward fibroblasts, smooth muscle cells, and glial cells [5]. PDGF has been implicated in the pathogenesis of angiogenesis, and it is widely used as a biomarker for tumor types of hepatic fibrosis, liver cancer, and gastrointestinal stromal tumors [6]. Thus, the precise and sensitive evaluation of these proteins in biological samples will be substantial for disease diagnosis and biomedical applications. The gold standard method for the detection of PDGF and thrombin is enzyme-linked immunosorbent assay (ELISA), which involves antibodies and an enzyme label [7,8]. However, the utilization of antibodies may encounter some drawbacks with their production, stability, and modification. 

Aptamers are single-stranded DNA (ssDNA), RNA, or modified nucleic acids that are obtained via an in vitro process called SELEX (systematic evolution of ligands by exponential enrichment) [9,10]. Since aptamers were first discovered in 1990, many aptamers have been identified toward a wide range of targets, such as metal ions [11], organic molecules [12], peptides [13], proteins [14,15,16,17], and whole cells [18]. Compared with antibodies acting as recognition elements, aptamers can retain high binding affinity to their targets under a wide range of conditions while possessing significant advantages such as easy synthesis, design flexibility, and desirable stability [19]. Nowadays, aptamers are identified for PDGF-BB and thrombin, and are used to develop aptasensors for the individual detection of PDGF-BB and thrombin in connection with different transducers, such as fluorescence [20,21], chemiluminescence [22,23], colorimetry [24], and electrochemistry [25,26]. The simultaneous detection of PDGF-BB and thrombin using aptamers as bioaffinity agents has been reported [27,28,29]. However, the majority of those reported aptasensors require trained personnel, expensive instrumentation, and are often laboratory-based, limiting their use at point-of-need and point-of-care settings.

In recent years, lateral flow aptasensors (LFAs) have garnered increasing interest as they offer a highly cost-effective and more flexible alternative to antibodies [30]. LFAs have been reported for the detection of various targets including toxins [31,32,33], proteins [1,34,35], and cancer cells [36]. Our group and others developed LFAs for the detection of thrombin [1,35]. However, to the best of our knowledge, the simultaneous detection of PDGF and thrombin using an LFA has not been reported. In the present work, we first report an LFA for the simultaneous visual detection of PDGF-BB and thrombin. The assay involved the capture of PDGF-BB and thrombin in sandwich-type formats between the capture aptamers on the test zones of LFA and gold nanoparticle (AuNP)-labeled detection aptamers. AuNPs were thus captured on the different test zones of the LFA and gave red bands to enable the visual detection of target proteins. Quantitative detection can be achieved by using a portable ‘strip reader’ with ‘Gold-Bio strip reader’ software. The LFA was used successfully for the detection of PDGF-BB and thrombin in human serum samples. 

## 2. Results and Discussion

**Figure 1:**
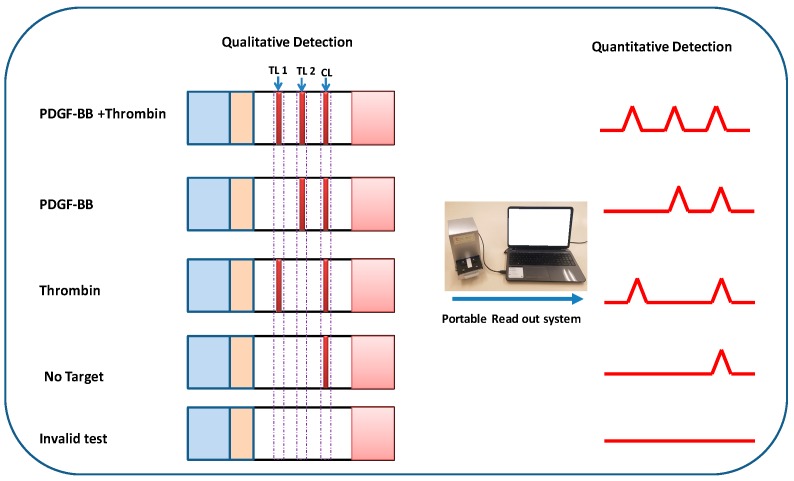
Principle of qualitative and quantitative detection of platelet-derived growth factor-BB (PDGF-BB) and thrombin on lateral flow aptasensors. TL: test line; CL: control line.

**Figure 2:**
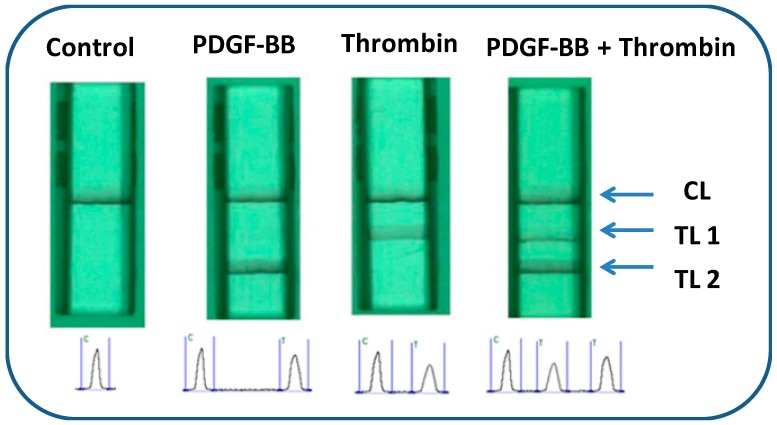
Typical photo images of lateral flow aptasensors (LFAs) and recorded responses of the LFAs in the presence of 0 nM thrombin + 0 nM PDGF-BB (control); 50 nM PDGF-BB; 50 nM thrombin; and 50 nM thrombin + 50 nM PDGF-BB. The optical intensities of the test and the control lines were recorded simultaneously using ‘Gold-Bio strip reader’ software, which could search the red bands in a fixed reaction area automatically and then determine parameters such as peak area integral. TL1: test line 1; TL2: test line 2; CL: control line. Please confirm if it’s necessary to explain C and T in the below of this figure.

The lateral flow aptasensor assay involved the capture of PDGF-BB and thrombin simultaneously in sandwich-type formats between the capture aptamers pre-immobilized on the test zones of LFA and AuNP-labeled detection aptamers on the conjugate pad. Typically, the sample solution containing PDGF-BB and thrombin was applied to the sample pad. Subsequently, the solution migrated by capillary action and rehydrated the AuNP-detection aptamer conjugates on the conjugate pad. Detection aptamers bind with targets and form PDGF-BB-aptamer-AuNP and thrombin-aptamer-AuNP complexes. Upon reaching the test zones, the complexes were captured by their respective capture aptamers immobilized on the test zones, giving two red bands (Figure 1). The capillary action caused the liquid sample to migrate further. Once the solution passed through the test zones, excess gold NP-detection aptamer conjugates were captured on the control zone via the hybridization events between the control DNA probes (pre-immobilized on the control zone) and the capture aptamers, thus forming a third red band. In the absence of targets, only one red band was observed in the control zone and no red band was observed in the test zones. In this case, the red band in the control zone (control line) showed that the LFA was working properly. For the control zone, we designed a DNA probe with a sequence complementary to the capture aptamer sequencesof PDGF-BB and thrombin. Qualitative analysis was simply performed by observing the color change of the test zones, and quantitative analysis was realized by reading the optical intensities of the red bands with a portable strip reader (Figure 1). The peak areas were proportional to the amounts of captured AuNPs on the test zones, which were proportional to the concentrations of PDGF-BB and thrombin in the sample solution. Figure 2 displays typical photo images and the corresponding responses of LFAs in the presence of (A) 0 nM PDGF-BB + 0 nM thrombin, (B) 50 nM PDGF-BB, (C) 50 nM thrombin, and (D) 50 nM PDGF-BB + 50 nM thrombin. One can see that PDGF-BB and thrombin do not cause interference for each other and can be detected simultaneously. 

### 2.1. Optimization of Experimental Parameters

**Figure 3:**
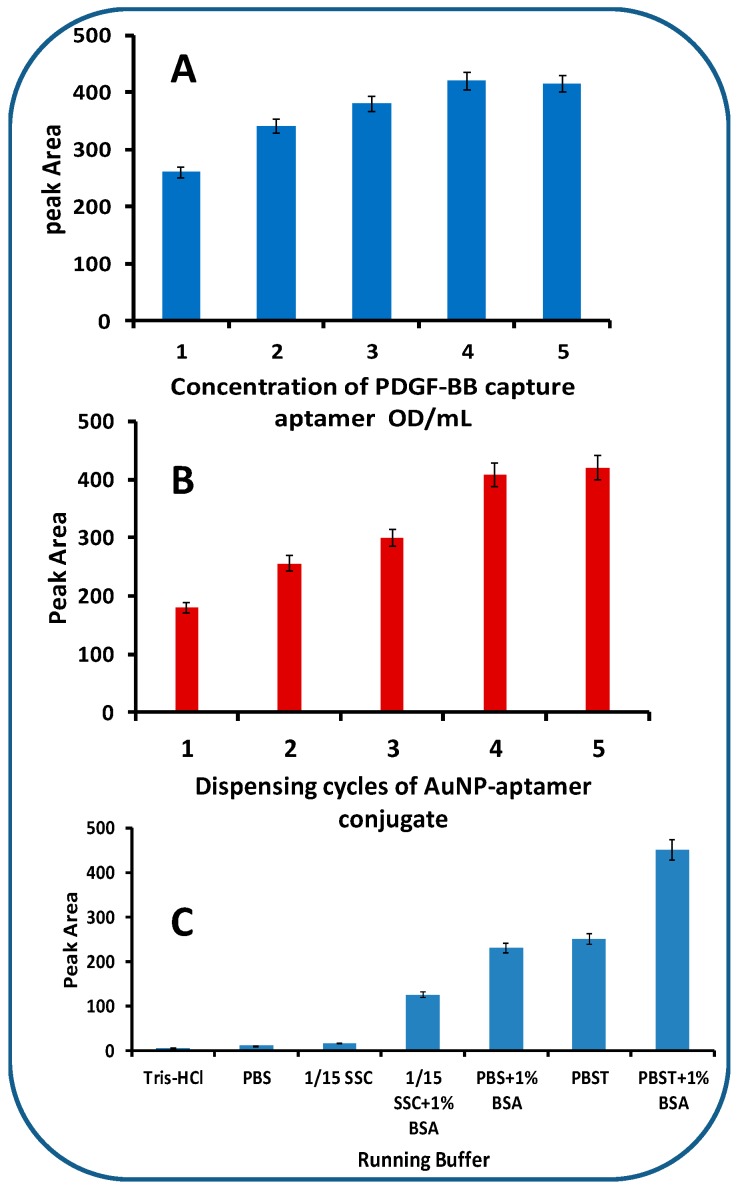
(**A**) Effect of PDGF-BB capture aptamer concentration on the response of LFA. PDGF-BB concentration: 25 nM; assay time: 10 min; sample solutions were prepared with PBST + 1% BSA; dispensing cycles of gold nanoparticle (AuNP)-aptamer conjugate on the conjugate pad: 4. (**B**) Effect of dispensing cycles of AuNP-aptamer conjugate on the response of LFA. PDGF-BB concentration: 25 nM; Assay time: 10 min; sample solution was prepared with PBST + 1% BSA; concentration of capture aptamer for the preparation of PDGF-BB test line: 4 OD/mL. (**C**) Effect of running buffer components on the response of LFA. PDGF-BB concentration: 25 nM; assay time: 10 min; concentration of capture aptamer for the preparation of PDGF-BB test line: 4 OD/mL; dispensing cycles of AuNP-aptamer conjugate: 4.

The analytical parameters, including the concentrations of capture aptamers on the test zones, the dispensing cycles of the AuNP-aptamer conjugates, and the components of the running buffers, were optimized using PDGF-BB and its aptamers as a model. The intensity of the LFA test zone was affected by the concentration of the capture aptamer (Figure 3A). One can see that the intensity of the LFA test zone increased with the increase of capture aptamer concentration from 1 OD/mL to 4 OD/mL; a further concentration increase resulted in signal saturation. As a result, 4 OD/mL of captured aptamer was used to prepare the test zone. The volume of AuNP-aptamer conjugates loaded on the conjugate pad affected the intensities of both test and control zones greatly. To obtain a maximum response using a minimal amount of AuNP-aptamer conjugates, the amount of AuNP-aptamer conjugates on the conjugate pad was optimized by increasing the dispensing cycles of the conjugate solutions. Figure 3B displays the histogram for the peak area of test line with an increasing cycle. It can be seen that the peak area from the test line of LFA increased up to four cycles. Therefore, a four-cycle procedure was employed as the optimal dispensing cycle in the following experiments. 

Four kinds of buffers were used for the fabrication and assay of the LFA: (1) 0.05 M Tris-HCl buffer containing 0.25% Triton X-100, 5% Tween, and 0.15 M NaCl (pH 8.0) was used to treat the sample pad. This treatment facilitates the transportation and reduces the amount of PDGF-BB and thrombin trapped on the sample pad. (2) A buffer containing 20 mM Na_3_PO_4_, 5% BSA, 0.25% Tween, and 10% sucrose was used to disperse the pellet of the AuNP-aptamer conjugates. This buffer stabilizes the nanoparticles, reduces the nonspecific adsorption of conjugates on the nitrocellulose pad, and facilitates the release of the conjugates from the conjugate pad. (3) PBS (0.01 M, pH 7.4) was used to prepare the streptavidin-biotinylated capture aptamer complexes. (4) Running buffer was used to prepare the sample solutions and to wash the LFA during the assay. The running buffer’s composition had a significant effect on the performance of the biosensor. Several buffers, including Tris-HCL, PBS, 1/15 SSC, 1/15 SSC (1% BSA), PBS (1% BSA), PBST, and PBST (1% BSA) were tested, and the results are shown in Figure 3C. The best response was obtained with the PBST (1% BSA) buffer. Thus, PBST (1% BSA) was chosen as the running buffer for the entire study.

### 2.2. Analytical Performances

**Figure 4:**
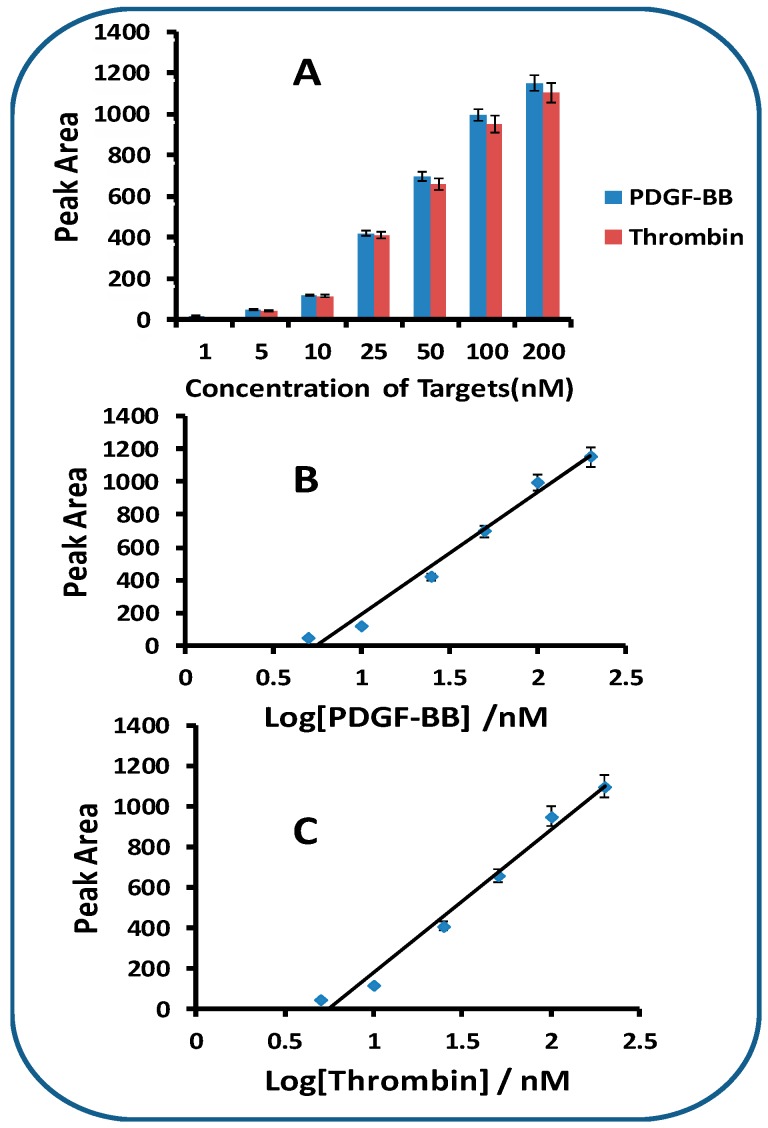
(**A**) Response histogram of the LFA test lines in the presence of thrombin and PDGF-BB at different concentrations. Assay time: 10 min; concentration of capture aptamers for the preparation of test lines: 4 OD/mL; cycles of dispensing AuNP-aptamer conjugates: 4; sample solutions were prepared with PBST + 1% BSA. (**B**) Calibration curve of the LFA for the detection of PDGF-BB. (**C**) Calibration curve of the LFA with for the detection of thrombin.

Under optimal experimental conditions, we examined the performance of the LFA with the samples containing different concentrations of PDGF-BB and thrombin. The intensities of the test zones were recorded with a portable strip reader and plotted as a function of different concentrations of PDGF-BB and thrombin (Figure 4). One can see that the peak areas of test lines increased with the increases of both PDGF-BB and thrombin concentrations (Figure 4A). Linear relationships between the peak areas and the logarithm of the target concentrations were obtained in the range of 1 nM–200 nM for both PDGF-BB (Figure 4B) and thrombin (Figure 4C). The detection limits were estimated to be 1.0 nM for PDGF-BB and 1.5 nM for thrombin (signal to noise (S/N) equals 3). The detection limits are comparable with those obtained with fluorescent aptasensors [20,21] and colorimetric aptasensors [24], and higher than those obtained with electrochemical aptasensors [25,26] and chemiluminescent aptasensors [22,23]. The signals of the LFA were saturated when the concentrations of PDGF-BB and thrombin were more than 250 nM. The specificity of LFA was assessed by testing the responses of other proteins (HSA, casein, BSA, IgG, and IgM) at 250 nM, as well as the mixture of target proteins (25 nM for both PDGF-BB and thrombin) and non-target protein (250 nM). Negligible signals were obtained in the absence of PDGF-BB and thrombin, and in the presence of 250 nM of non-target protein (results not shown). It is noted that the responses of target proteins (25 nM) were not affected in the presence of high concentrations of non-target proteins (250 nM). Therefore, the LFA shows excellent specificity for both PDGF-BB and thrombin. Since quantitative analysis relies on the stability of the analyte signal and the reproducibility of the assay, six LFAs were tested with 25 nM of PDGF-BB and thrombin to determine the reproducibility of the LFA. The relative standard deviation (RSD) for PDGF-BB and thrombin were 8.9% and 8.6%, respectively, indicating a sufficient reproducibility. 

### 2.3. Detecting PDGF -BB and Thrombin in Human Serum

**Figure 5:**
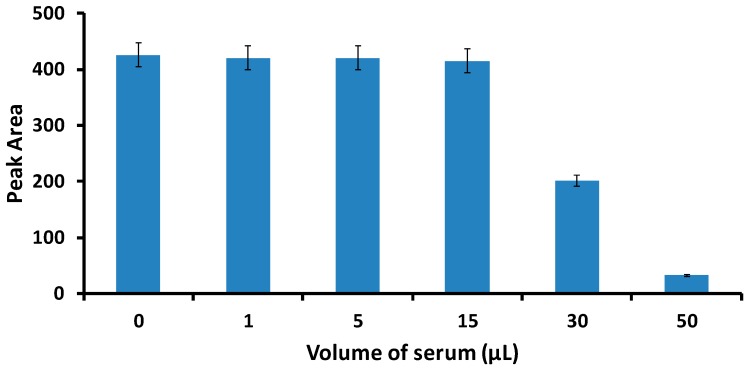
Effect of the spiked serum volume on the response of the LFA. The concentration of thrombin is 25 nM. Other experimental conditions were the same as that described in Figure 4.

The LFA was also successfully applied to detect PDGF-BB and thrombin in spiked human serum samples. The sample solutions were prepared by spiking different concentrations of standard PDGF-BB and thrombin solution into the serum. We studied the matrix effect of serum on the signals of LFA test lines by using different volumes of serum (1 to 50 μL). It was found that there was no matrix effect observed when the serum volume was less than 15 μL (Figure 5). Thereby, 15 μL of serum was applied in the following experiments. The serum samples spiked with different concentrations of PDGF-BB and thrombin were prepared to obtain the calibration curves in a complex biological matrix. The tests were performed under the best experimental conditions, as shown in Figure 5. Each sample was tested three times with three LFAs and the average responses were used to plot the calibration curves of PDGF-BB and thrombin. The response histogram of the LFA test lines in the presence of thrombin and PDGF-BB in spiked serum and corresponding calibration curves is similar to that shown in Figure 4 (results not shown). Detection limits of 1 nM for PDGF-BB and 2 nM for thrombin were obtained (S/N = 3).

## 3. Experimental Section

### 3.1. Apparatus

The Airjet AJQ 3000 dispenser, Biojet BJQ 3000 dispenser, clamshell laminator, and guillotine cutting module CM 4000 were from Biodot LTD (Irvine, CA, USA). The portable strip reader, DT1030, was purchased from Shanghai Goldbio Tech. Co., LTD (Shanghai, China).

### 3.2. Reagents and Materials

Glass fibers (GFCP000800), cellulose fiber sample pads (CFSP001700), laminated cards (HF000MC100), and nitrocellulose membranes (HFB24004) were purchased from Millipore (Bedford, MA, USA). Streptavidin from *Streptomyces avidinii*, dithiothreitol (DTT), triethylamine (TEA), ethyl acetate, Na_3_PO_4_·12H_2_O, HAuCl_4_, trisodium citrate, sucrose, Tween 20, Triton X-100, sodium chloride-sodium citrate (SSC) buffer (pH 7.0), phosphate buffer saline (PBS, PH 7.4, 0.01 M), bovine serum albumin (BSA), human serum albumin (HSA), thrombin (from human plasma), and PDGF-BB were purchased from Sigma-Aldrich (St. Louis, MO, USA)). The aptamers and oligonucleotide probes used in this study were obtained from Integrated DNA Technologies, Inc. (Coralville, IA, USA) and have the following sequences:

**Detection aptamer for thrombin:**
  5′-/5ThioMC6-D/TT TTT TTT TTT TTT TTT TTT GGT TGG TGT GGT TGG-3′;
**Capture aptamer for thrombin:**
  5′-AGT CCG TGG TAG GGC AGG TTG GGG TGA CT-/3BioTEG/-3′;
**Detection aptamer for PDGF-BB:**
  5′-/5ThioMC6-D/TAC TCA GGG CAC TGC AAG CAA TTG TGG TCC CAA TGG GCT GAG TAT-3′;
**Capture aptamer for PDGF-BB:**
  5′- TAC TCA GGG CAC TGC AAG CAA TTG TGG TCC CAA TGG GCT GAG TA/3BioTEG/-3′;
**DNA oligonucleotide (control probe):**
  5′-ATA CTC AGC CAA TTG GGA CCA CAA TTG CTT GCA GTG CCC TGA GTA AAA AAA AAA AAA AAA AAA AA-biotin-3′.

All other chemicals were of analytical reagent grade. All buffer solutions were prepared using ultrapure (>18 MΩ cm) water from a Millipore Milli-Q water purification system (Billerica, MA, USA).

### 3.3. Preparation of Gold Nanoparticles (AuNPs)

Citrate reduction of HAuCl_4_ was used to synthesize AuNPs with an approximate diameter of 15 ± 3.5 nm [1]. All glassware used in this preparation was thoroughly cleaned in aqua regia (three parts HCl/one part HNO_3_), rinsed with double distilled H_2_O, and oven-dried prior to use. A 250-mL aqueous solution of 0.01% HAuCl_4_ was heated to boiling and vigorously stirred in a 500-mL round-bottom flask; 4.5 mL of 1% trisodium citrate was added quickly to this solution. The color changed from deep blue to wine red in about 60 s. Boiling was continued for an additional 10 min. The solution was cooled to room temperature with a continuous stirring for another 15 min. The AuNPs were stored in dark bottles at 4 °C. 

### 3.4. Preparation of AuNP-Aptamer Conjugates

Thiolated aptamers (detection aptamers) were used for conjugation with AuNPs. The aptamers were first activated following the procedure: 100 μL of thiolated aptamer (1.0 OD) was mixed with 2 μL of TEA and 7.7 mg of DTT to react for 1 h at room temperature, then the excess DTT was removed by extraction with 400 μL of ethyl acetate solution. The activated aptamer was then added to 1 mL of 5-fold concentrated AuNP solution. After standing for 24 h, the conjugate was aged with the addition of PBS until a final concentration of 0.01 M was reached. The solution could stand for another 24 h at 4 °C, followed by centrifugation for 20 min at 12,000 rpm to remove the excess reagents. After discarding the supernatant, the red pellets were washed, recentrifuged, and resuspended in 1 mL of an aqueous solution containing 20 mM Na_3_PO_4_·12H_2_O, 5% BSA, 0.25% Tween 20, and 10% sucrose.

### 3.5. Preparation of Lateral Flow Aptasensors (LFAs)

**Figure 6:**
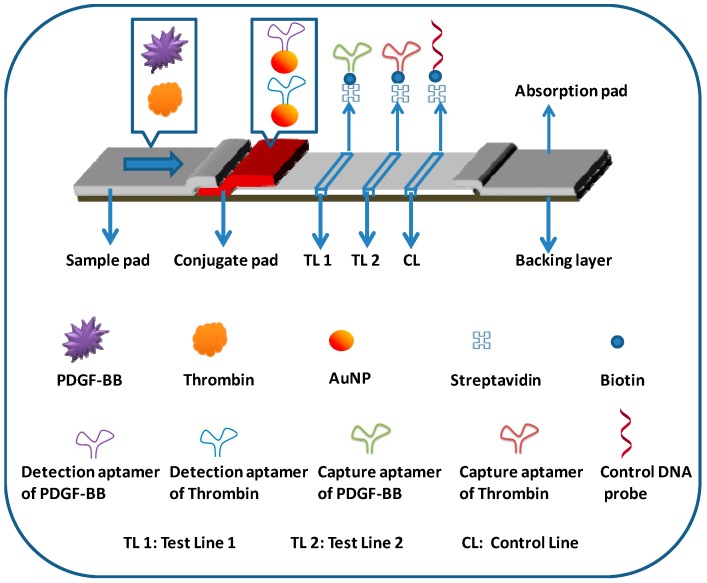
Schematic representation of lateral flow aptasensor for the simultaneous detection of PDGF-BB and thrombin.

An LFA consists of four components: sample application pad, conjugate pad, nitrocellulose membrane, and absorption pad (Figure 6). The components were mounted on a common backing layer (typically, an inert plastic, e.g., polyester). Two pairs of aptamers that bind to PDGF-BB and thrombin, respectively, were used to prepare the LFA. Test zones were prepared by dispensing the streptavidin-biotinylated capture aptamers of PDGF-BB and thrombin onto the nitrocellulose membrane at different locations. The detection aptamers of PDGF-BB and thrombin were labeled with AuNPs, the conjugates were mixed at 1:1 ratio (volume) and dispensed on the conjugate pad. The sample application pad (17 mm × 30 cm) was made from cellulose fiber (CFSP001700, Millipore) and was soaked with a buffer (pH 8.0) containing 0.25% Triton X-100, 0.05 M Tris-HCl, and 0.15 mM NaCl. Then it was dried and stored in a desiccator at room temperature. The conjugate pad was prepared by dispensing a desired volume of AuNP-aptamer conjugate solution onto the glass fiber pad (8 mm × 30 cm) using an Airjet AJQ 3000 dispenser. The pad was dried at room temperature and stored in a desiccator at 4 °C. The test zone and control zone on the nitrocellulose membrane (25 mm × 30 mm) were prepared by dispensing the capture aptamer and biotinylated DNA (control probe, complementary with one of the detection aptamers) solutions, respectively. The distance between each zone was around 2 mm and there were a total of three zones (test zone 1 for PDGF-BB; test zone 2 for thrombin; and control zone) on the biosensor. To facilitate the immobilization of the probes, streptavidin was used to react with the biotinylated aptamers (capture aptamers) and the biotinylated control DNA probe. Briefly, 42 μL of 10 OD biotinylated aptamer was mixed with 200 μL of 2 mg/mL streptavidin. After incubating for 1 h at room temperature, 508 μL of PBS was added to the mixture. The excess aptamer was removed by centrifuge for 30 min with a filter (cutoff 30,000, Millipore) at 6000 rpm. The conjugate was washed twice with 500 μL of PBS in the same centrifugal filter. The remaining solution in the filter was collected, and the solution was diluted to 500 μL by adding PBS. Following the same procedure, the biotinylated DNA with a concentration of 10 OD was used to prepare the streptavidin-biotinylated DNA complex for the control zone. The streptavidin-biotinylated aptamer and streptavidin-biotinylated DNA were dispensed on the nitrocellulose membrane to form the test zones and control zone, respectively. The membrane was dried at room temperature for 1 h and stored at 4 °C. Finally, all the parts were assembled on a plastic adhesive backing layer (typically, an inert plastic, e.g., polyester) using the clamshell laminator (Biodot, CA). Each part overlapped 2 mm to ensure that the solution migrated through the LFA during the assay. LFAs with a 3-mm width were cut using the guillotine cutting module CM 4000.

### 3.6. Assay Procedure

A sample solution of 80 μL containing the desired concentration of PDGF-BB and thrombin in the running buffer (PBST containing 1% BSA) was applied to the sample pad. The test zone and control zone were evaluated visually within 10 min (qualitative measurement). For quantitative measurements, the optical intensities of the red bands were recorded with a portable ‘strip reader’. For detecting PDGF-BB and thrombin in serum, sample solutions were prepared by mixing 65 μL of running buffer with 15 μL of plasma, which were spiked with different quantities of PDGF-BB and thrombin, and then the sample solution was applied to the sample pad. Qualitative and quantitative measurements were the same as described above.

## 4. Conclusions

We have developed a lateral flow aptasensor (LFA) for the simultaneous detection of PDGF-BB and thrombin in a complex biological matrix. The performance of the LFA shows great promise for the development of aptamer-based lateral flow strip biosensors for point-of-care or for the in-field detection of disease-related protein biomarkers. The multiplex capability of the proposed LFA can be realized with an LFA array by spotting multiple capture aptamers on the test zone. Future work will aim to improve the sensitivity of the LFA and apply it for the detection of cancer protein biomarkers.
